# Image quality comparison of AirDoc portable retina camera versus eyer in a diabetic retinopathy screening program

**DOI:** 10.1186/s40942-024-00559-z

**Published:** 2024-06-14

**Authors:** Rodrigo Brant, Luis Filipe Nakayama, Talita Virgínia Fernandes de Oliveira, Juliana Angelica Estevão de Oliveira, Lucas Zago Ribeiro, Gabriela Dalmedico Richter, Rafael Rodacki, Fernando Marcondes Penha

**Affiliations:** 1 Ophthalmology and Visual Science Department, Sao Paulo Federal University, Sao Paulo, SP Brazil; 2 Keck School of Medicine, Roski Eye Institute, University of Southern California, Los Angeles, USA; 3 Laboratory for Computational Physiology, Massachusetts Insitute of Technology, Cambridge, MA USA; 4 Fundação Universidade Regional de Blumenau, Blumenau, SC Brazil

**Keywords:** Retina, Diabetic retinopathy, Portable retinal cameras, Image quality

## Abstract

**Background:**

Diabetic retinopathy (DR) stands as the foremost cause of preventable blindness in adults. Despite efforts to expand DR screening coverage in the Brazilian public healthcare system, challenges persist due to various factors including social, medical, and financial constraints. Our objective was to evaluate the quality of images obtained with the AirDoc, a novel device, compared to Eyer portable camera which has already been clinically validated.

**Methods:**

Images were captured by two portable retinal devices: AirDoc and Eyer. The included patients had their fundus images obtained in a screening program conducted in Blumenau, Santa Catarina. Two retina specialists independently assessed image’s quality. A comparison was performed between both devices regarding image quality and the presence of artifacts.

**Results:**

The analysis included 129 patients (mean age of 61 years), with 29 (43.28%) male and an average disease duration of 11.1 ± 8 years. In Ardoc, 21 (16.28%) images were classified as poor quality, with 88 (68%) presenting artifacts; in Eyer, 4 (3.1%) images were classified as poor quality, with 94 (72.87%) presenting artifacts.

**Conclusions:**

Although both Eyer and AirDoc devices show potential as screening tools, the AirDoc images displayed higher rates of ungradable and low-quality images, that may directly affect the DR and DME grading. We must acknowledge the limitations of our study, including the relatively small sample size. Therefore, the interpretations of our analyses should be approached with caution, and further investigations with larger patient cohorts are warranted to validate our findings.

## To the editor

Portable retinal cameras represent cost-effective, portable, and technically simpler alternative devices for diabetic retinopathy screening, with comparable performance to tabletop cameras [1–4], potentially increasing screening coverage and enabling early diagnosis and treatment.

In light of these considerations, this study aims to evaluate the quality of images obtained with the AirDoc, a novel device, compared to Eyer portable camera, which has already been clinically validated [5, 6].

This cross-sectional study included Brazilian patients from the Diabetic Retinopathy (DR) Screening Program, “Mutirão do Diabetes,” conducted in Blumenau, Santa Catarina, Brazil, in the year 2022. The study was conducted following the principles of the Helsinki Declaration and was approved by the research ethics committee of the Regional University of Blumenau, FURB, Blumenau, Santa Catarina, Brazil (CAAE 64797822.6.0000.5370).

All participants provided and signed an informed consent form. Patients over 18 years old with type 1 or type 2 diabetes who agreed to participate in the study were included, while patients with contraindications for pharmacological mydriasis were excluded.

All images were captured after pharmacological mydriasis with 0.5% tropicamide eye drops, instilled three times in each eye by two healthcare professionals (TVFO and JAEO), familiar with portable camera capturing process, and with similar training. This study included a single retinography image centered on each patient’s macular area of each eye [6]. All images and tabular data were anonymized and manually reviewed to ensure the absence of sensitive data that could lead to identification.

The study included two portable cameras: the AirDoc Fundus Camera and the Eyer Phelcom.

Airdoc Technology (AirDoc, Beijing, China) is a medical and AI technology company founded in 2015 in China. It specializes in AI software for assessing the risk of chronic diseases based on fundus images and the development of digital retinal cameras. The portable digital retinal camera represented by the brand is the AI-FD16aF, weighing 1.5 kg and measuring 280 × 240 × 130 mm (LxWxH). The retinal camera features voice commands, and a 40-degree field of view, and captures images in less than a minute.

The Eyer (Phelcom Technologies, Sao Carlos, Brazil) is a retinal camera mounted on a Samsung Galaxy S10 smartphone (Android 11). The camera captures retinal images at a 45-degree angle, utilizes a 12-megapixel sensor, produces images of 1600 × 1600 pixels, and has an autofocus control that ranges from − 20 to + 20 diopters.

Demographic data, including gender and age, as well as clinical data, such as diabetes duration, insulin use, and comorbidities, were collected during the project.

Image quality was classified as acceptable or unacceptable in cases where it was not possible to assess at least 2/3 of the image clearly. Image artifacts were considered present when any image artifact, such as lighting alterations, dust, or loss of focus, was visible in the photograph, even if it allowed for image assessment [7]. In image quality and artifact criteria, a single evaluator judgment was required for quality labeling.

Statistical analysis involved the comparison of demographic data, image quality and presence of artifacts, between the AirDoc and the Eyer retinal cameras.

Continuous variables were presented with mean and standard deviation, while categorical variables were presented with counts and percentages. Mann-Whitney was applied to compare continuous variables, Chi-square and McNemar’s tests were used to compare categorical variables, and weighted Cohen’s Kappa test was used to compare image quality between devices. Statistical tests were conducted using Python 3.10 and packages. A significance level of 0.05 was used to define statistical significance.

In the present study, 129 retinal photographs from 67 patients were included. The mean age of the included patients was 61 ± 11.15 years, with 38 (56.72%) female patients. The mean duration of diabetes since diagnosis was 11.1 ± 8 years. The study included 64 right eyes (49.6%) and 65 left eyes (50.4%).

**Fig. 1:**
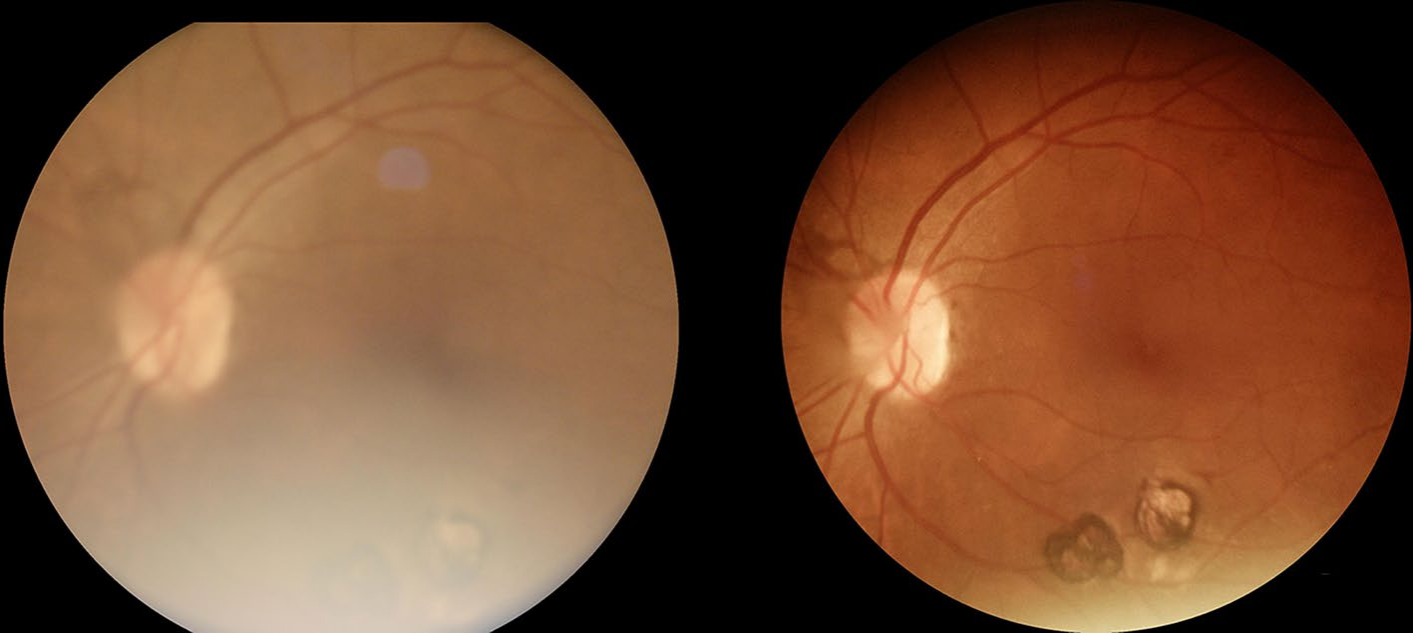
AirDoc color fundus photograph (left) compared to the respective Eyer color fundus photograph (right), depicting an example of lower quality obtained with the AirDoc device

**Fig. 2:**
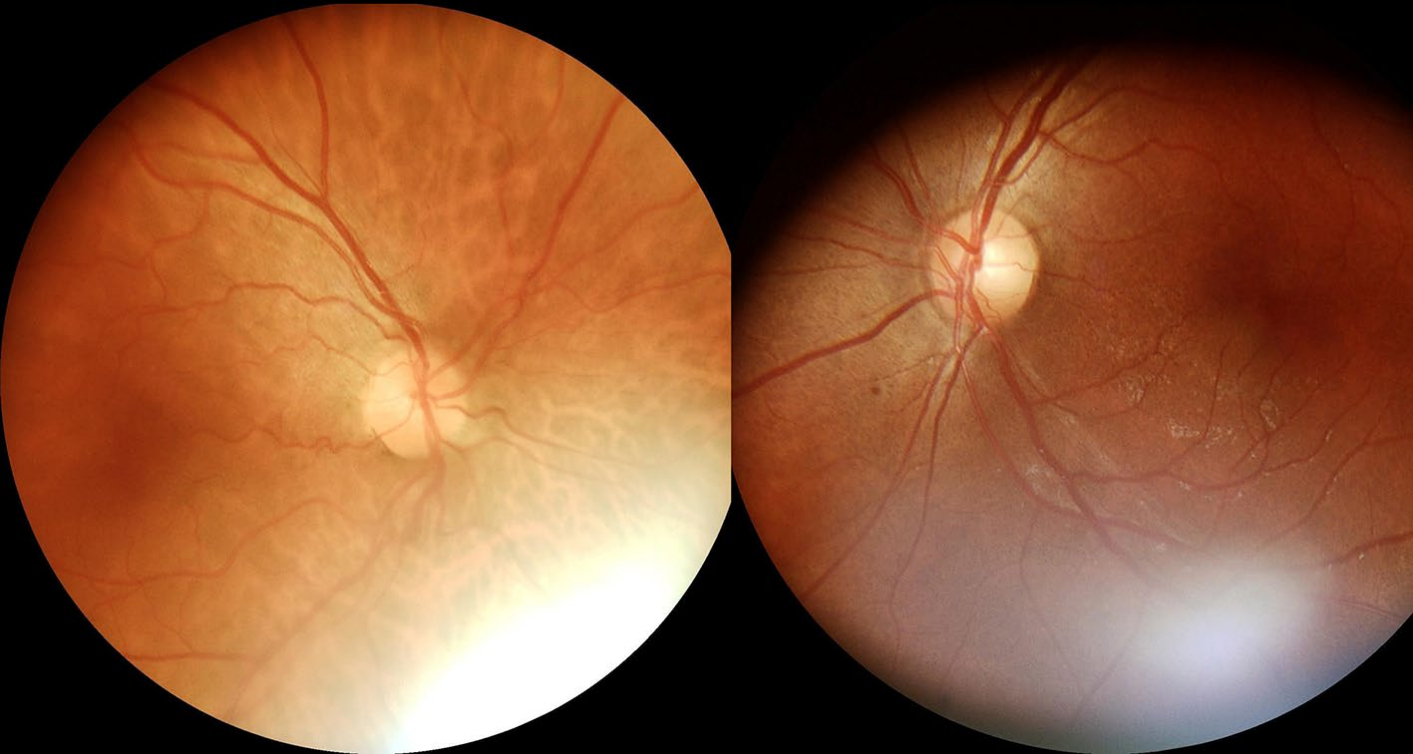
Examples of color fundus photographs with image artifacts; in such cases, despite the presence of artifacts, image grading was performed as per the study criteria

**Fig. 3:**
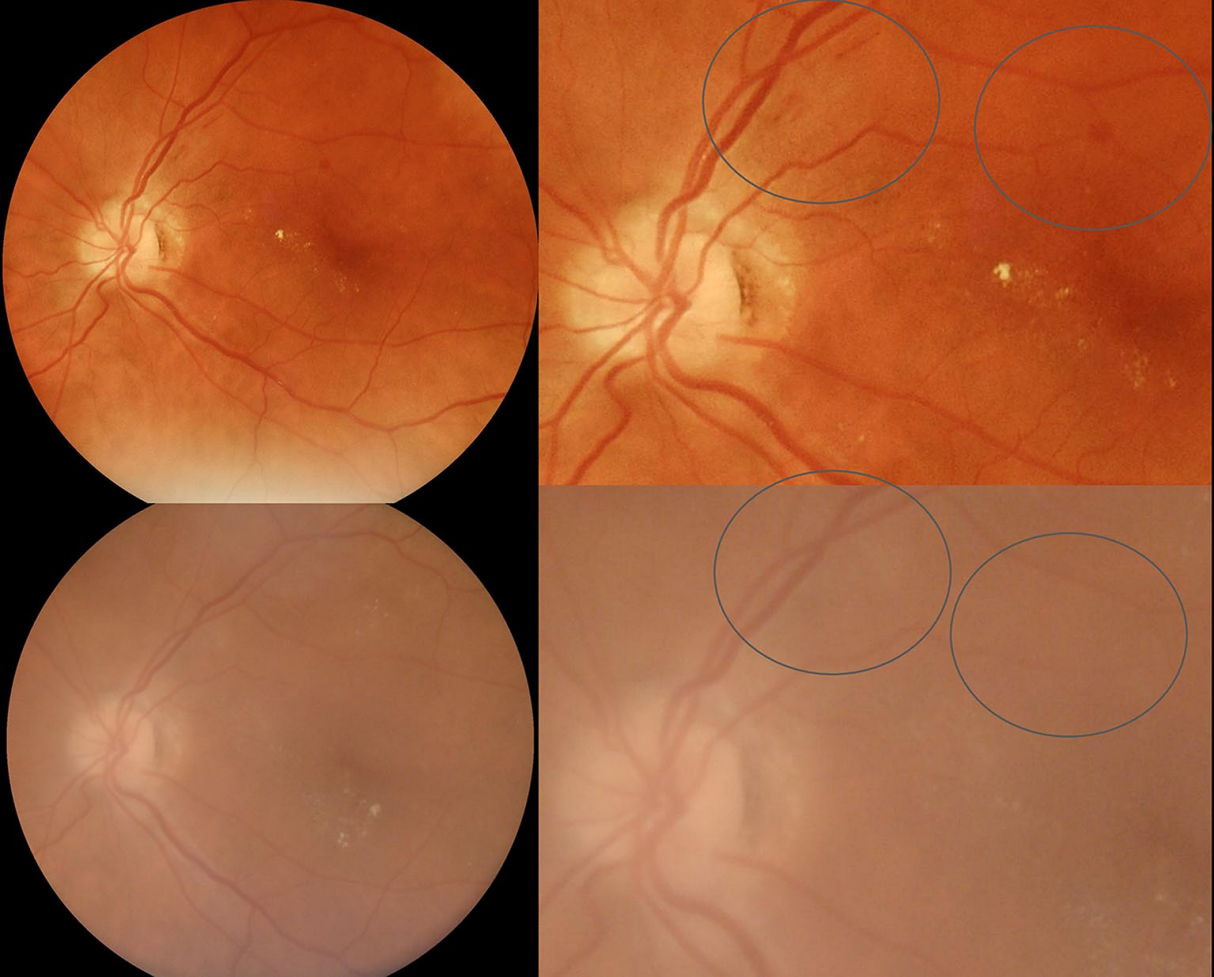
Comparative montage of images obtained with Eyer (upper images) and AirDoc (lower images). In this example, hemorrhages are not clearly identified in AirDoc due to poor image quality

In examinations conducted with the Eyer retinal camera, 4 (3.1%) images were classified by the human readers as having insufficient quality for analysis, whereas with the AirDoc camera, 21 (16.28%) images were classified as having insufficient quality, showing a statistically significant difference between the cameras (McNemar 23.04; *P* < .001). (Figures 1, 2 and 3; Table 1)

**Table 1:** Comparison of image quality between AirDoc and Eyer

	Airdoc	Yes	No	Total
*Eyer*	Yes	105	20	125
	No	3	1	4
Total		108	21	

In examinations conducted with the Eyer camera, 94 images (72.87%) exhibited some form of artifact, while with the AirDoc camera, 88 images (68.21%) had artifacts, also demonstrating a statistically significant difference between the cameras (McNemar 96.5; *P* < .001) (Table 2).

**Table 2:** Comparison of image artifacts between AirDoc and Eyer

	Airdoc	Yes	No	Total
*Eyer*	Yes	73	31	104
	No	25	13	48
Total		98	44	

For diabetic retinopathy grading, the AirDoc camera presented an overall sensitivity of 50.3% and an overall specificity of 81.8%, compared to the Eyer retinal camera, with lower sensitivity for mild non-proliferative DR (28.6%) and lower specificity for normal images (61.5%).

The sensitivity analysis conducted to evaluate the influence of demographics on image quality revealed no significant difference in gender distribution regarding image quality. Age was statistically related to poor quality. (Table 3)

**Table 3:** Demographics and clinical differences regarding quality

Quality		No		Yes		*P*
Gender						
	*Male*	7	66.67%	50	46.30%	0.274
	*Female*	14	33.33%	58	53.70%	
**Age (mean, SD)**		63.85	10.791	58.78	11.21	0.04

In our analysis, we observed that the Eyer images exhibited a higher frequency of image artifacts, while the AirDoc images displayed more ungradable images. An illumination issue was detected in 75.20% of the Eyer images, while 7.20% exhibited focus problems. Conversely, among Airdoc images, 40.74% showed illumination problems, and 20.37% focus issues. This contrasts with previous studies utilizing other handheld devices, which reported higher gradability rates [8–10]. The lower gradability rates observed in our study could be attributed to potentially more stringent criteria for assessing photo quality and unfamiliarity with the AirDoc device [4]. The prevalence of low-quality images in the AirDoc camera within our study population suggests that the image quality of the Eyer system surpassed that of the AirDoc, may potentially contribute to false-negative rates in the assessment of retinal images.

High-quality images are crucial for effective retinal screening via retinal fundus photos. Various factors, including operator training, patient age, duration of diabetes, poor cooperation, mydriasis, and media opacity, can significantly influence image quality [11–13]. Furthermore, suboptimal image quality may result in an increased number of referable cases and incorrect predictions by automated systems [14]. A high rate of ungradable images is known to negatively impact the efficacy of a DR screening program in a significant manner [15].

We must acknowledge the limitations of our study, including the relatively small sample size of 67 patients and 129 eyes. Lack of a regular ophthalmic evaluation to address the reasons for poor-quality pictures in both groups, such as cataracts, poor dilation, and other media opacities. Therefore, the interpretations of our analyses should be approached with caution, and further investigations with larger patient cohorts are warranted to validate our findings. Healthcare professionals exhibited more familiarity with the Eyer camera, potentially influencing the image-capturing process. The present paper did not address the predictive ability of demographic or clinical characteristics for referable DR.

In conclusion, the AirDoc device demonstrates potential as a screening tool, but in our studied population, the images exhibit lower image quality that may impact in the diabetic retinopathy grading.

## Data Availability

No datasets were generated or analysed during the current study.

## Declarations

### Human accordance Statement

All procedures performed in studies involving human participants were in accordance with the ethical standards of the institutional and/or national research committee and with the 1964 Helsinki Declaration and its later amendments or comparable ethical standards. The study was approved by the Bioethics Committee of the Regional University of Blumenau, FURB, Blumenau, Santa Catarina, Brazil (CAAE 64797822.6.0000.5370).

## Abbreviations

DR: Diabetic Retinopathy

## References

[1] Salongcay RP, Aquino LAC, Salva CMG, Saunar AV, Alog GP, Sun JK et al. Comparison of Handheld Retinal Imaging with ETDRS 7-Standard Field Photography for Diabetic Retinopathy and Diabetic Macular Edema. Ophthalmol Retina [Internet]. 2022;6(7):548–56. 10.1016/j.oret.2022.03.002. 10.1016/j.oret.2022.03.002 PMC12764358 35278726

[2] Piyasena MMPN, Yip JLY, MacLeod D, Kim M, Gudlavalleti VSM. Diagnostic test accuracy of diabetic retinopathy screening by physician graders using a hand-held non-mydriatic retinal camera at a tertiary level medical clinic. BMC Ophthalmol [Internet]. 2019;19(1):89. 10.1186/s12886-019-1092-3. 10.1186/s12886-019-1092-3 PMC6454614 30961576

[3] Midena E, Zennaro L, Lapo C, Torresin T, Midena G, Pilotto E et al. Handheld Fundus Camera for Diabetic Retinopathy Screening: A Comparison Study with Table-Top Fundus Camera in Real-Life Setting. J Clin Med Res [Internet]. 2022;11(9). 10.3390/jcm11092352. 10.3390/jcm11092352 PMC9103652 35566478

[4] de Oliveira JAE, Nakayama LF, Zago Ribeiro L, de Oliveira TVF, Choi SNJH, Neto EM et al. Clinical validation of a smartphone-based retinal camera for diabetic retinopathy screening. Acta Diabetol [Internet]. 2023;60(8):1075–81. 10.1007/s00592-023-02105-z. 10.1007/s00592-023-02105-z PMC10289975 37149834

[5] Nakayama LF, Zago Ribeiro L, Novaes F, Miyawaki IA, Miyawaki AE, de Oliveira JAE et al. Artificial intelligence for telemedicine diabetic retinopathy screening: a review. Ann Med [Internet]. 2023;55(2). 10.1080/07853890.2023.2258149. 10.1080/07853890.2023.2258149 PMC10515659 37734417

[6] Penha FM, Priotto BM, Hennig F, Przysiezny B, Wiethorn BA, Orsi J et al. Single retinal image for diabetic retinopathy screening: performance of a handheld device with embedded artificial intelligence. Int J Retina Vitreous [Internet]. 2023;9(1). 10.1186/s40942-023-00477-6. 10.1186/s40942-023-00477-6 PMC10332010 37430345

[7] Nakayama LF, Gonçalves MB, Ribeiro LZ, Malerbi FK, Regatieri CVS. Diabetic Retinopathy Labeling Protocol for the Brazilian Multilabel Ophthalmological Dataset [Internet]. 2023. 10.31219/osf.io/puznm.

[8] Zhang W, Nicholas P, Schuman SG, Allingham MJ, Faridi A, Suthar T et al. Screening for Diabetic Retinopathy Using a Portable, Noncontact, Nonmydriatic Handheld Retinal Camera. J Diabetes Sci Technol [Internet]. 2017;11(1):128–34. 10.1177/1932296816658902. 10.1177/1932296816658902 PMC5375071 27402242

[9] Caceres J, Zhang Y, Boe L, Zhou Y, Besirli C, Paulus YM et al. Diabetic Retinopathy Screening Using a Portable Retinal Camera in Vanuatu. Clin Ophthalmol [Internet]. 2023;17:2919–27. 10.2147/OPTH.S410425. 10.2147/OPTH.S410425 PMC10560479 37814638

[10] Jin K, Lu H, Su Z, Cheng C, Ye J, Qian D. Telemedicine screening of retinal diseases with a handheld portable non-mydriatic fundus camera. BMC Ophthalmol [Internet]. 2017;17(1):89. 10.1186/s12886-017-0484-5. 10.1186/s12886-017-0484-5 PMC5470179 28610611

[11] Li HK, Horton M, Bursell SE, Cavallerano J, Zimmer-Galler I, Tennant M et al. Telehealth practice recommendations for diabetic retinopathy, second edition. Telemed J E Health [Internet]. 2011;17(10):814–37. 10.1089/tmj.2011.0075. 10.1089/tmj.2011.0075 PMC6469533 21970573

[12] Davila JR, Sengupta SS, Niziol LM, Sindal MD, Besirli CG, Upadhyaya S et al. Predictors of Photographic Quality with a Handheld Nonmydriatic Fundus Camera Used for Screening of Vision-Threatening Diabetic Retinopathy. Ophthalmologica [Internet]. 2017;238(1–2):89–99. 10.1159/000475773. 10.1159/000475773 PMC5733780 28675903

[13] Malerbi FK, Andrade RE, Morales PH, Stuchi JA, Lencione D, de Paulo JV et al. Diabetic Retinopathy Screening Using Artificial Intelligence and Handheld Smartphone-Based Retinal Camera. J Diabetes Sci Technol [Internet]. 2021;1932296820985567. 10.1177/1932296820985567. 10.1177/1932296820985567 PMC9294565 33435711

[14] Ruamviboonsuk P, Tiwari R, Sayres R, Nganthavee V, Hemarat K, Kongprayoon A et al. Real-time diabetic retinopathy screening by deep learning in a multisite national screening programme: a prospective interventional cohort study. Lancet Digit Health [Internet]. 2022;4(4):e235–44. 10.1016/S2589-7500(22)00017-6. 10.1016/S2589-7500(22)00017-6 35272972

[15] Brennan IG, Kelly SR, McBride E, Garrahy D, Acheson R, Harmon J et al. Addressing Technical Failures in a Diabetic Retinopathy Screening Program. OPTH [Internet]. 2024 Feb 9 [cited 2024 Feb 12];18:431–40. https://www.dovepress.com/addressing-technical-failures-in-a-diabetic-retinopathy-screening-prog-peer-reviewed-fulltext-article-OPTH. 10.2147/OPTH.S442414 PMC10864767 38356695

